# LncRNA OIP5-AS1 loss-induced microRNA-410 accumulation regulates cell proliferation and apoptosis by targeting KLF10 via activating PTEN/PI3K/AKT pathway in multiple myeloma

**DOI:** 10.1038/cddis.2017.358

**Published:** 2017-08-10

**Authors:** Nan Yang, Jinqiu Chen, Hui Zhang, Xiaman Wang, Huan Yao, Yue Peng, Wanggang Zhang

**Affiliations:** 1 Department of Hematology, The Second Affiliated Hospital of Xi’an Jiaotong University, West Five Road, NO.157, Xi’an, China

## Abstract

Numerous studies confirmed that aberrant miRNAs expression contributes to multiple myeloma (MM) development and progression. However, the roles of specific miRNAs in MM remain to be investigated. In present study, we demonstrated that miR-410 expression was increased in MM newly diagnosed and relapsed tissues and cell lines. Clinical analysis revealed that miR-410 was positively correlated with advanced ISS stage. Moreover, high miR-410 expression in MM patients showed an obvious shorter overall survival and progression-free survival. Gain- and loss-of function experiments indicated that miR-410 promoted cell proliferation, cell cycle progression and apoptosis inhibition both *in vitro* and *in vivo*. Moreover, KLF10 was identified as a direct downstream target of miR-410 in MM cells, and mediated the functional influence of miR-410 in MM, resulting in PTEN/AKT activation. In clinical samples of MM, miR-410 inversely correlated with KLF10. Alteration of KLF10 expression or AKT inhibitor at least partially abolished the biological effects of miR-410 on MM cells. Furthermore, downregulated expression of lncRNA OIP5-AS1 was inversely correlated with miR-410 expression in MM tissues. LncRNA OIP5-AS1 could modulate the miR-410 expression and regulate its target KLF10/PTEN/AKT-mediated cellular behaviors. Taken together, this research supports the first evidence that lncRNA OIP5-AS1 loss-induced miR-410 accumulation facilitates cell proliferation, cycle progression and apoptosis inhibition by targeting KLF10 via activating PTEN/PI3K/AKT pathway in MM.

Multiple myeloma (MM) is an incurable plasma cell malignancy characterized by aberrant infiltration and accumulation of malignant plasma cells within the bone marrow (BM). It is the second most common hematologic neoplasms with dismal prognosis despite advanced progress in diagnosis and treatment, including immunomodulatory medicine, proteasome inhibitors and autologous stem cell transplantation.^1, 2, 3, 4, 5^ However, the detailed potential mechanisms for the development and progression of MM remain unclear. Therefore, it is urgent to elucidate the molecular mechanisms of MM and identify new prognostic biomarker to provide potential therapeutic targets for MM patients.

MicroRNAs (miRNAs), a class of endogenous evolutionarily conserved non-coding single-stranded small RNAs, act as post-transcriptional regulator of gene expression in cancer initiation, development and progression by interacting with complementary sequences within the 3′-untranslated region (UTR) of target mRNA to induce mRNA degradation or translational repression.^6, 7, 8, 9^ Increasing evidence confirm that aberrant miRNAs play critical roles in multiple biological processes in MM,^10, 11, 12^ including cell proliferation, apoptosis, drug-resistance, metastasis and stem cell renewal and have been identified as promising therapeutic and prognostic biomarkers in MM diagnosis and treatment.

MiR-410, a novel cancer-related microRNA, has been found to be dysregulated in cancers.^13, 14, 15^ Li *et al* demonstrated that miR-410 promotes cell proliferation by targeting BRD7 in non-small cell lung cancer (NSCLC).^16^ In prostate cancer, miR-410 could be served as a potential serum biomarker for the diagnosis.^17^ MiR-410 acts as oncogene in NSCLC through downregulating SLC34A2 via activating Wnt/*β*-catenin pathway.^18^ These reports identified miR-410 as an oncogene. However, miR-410 functions as a tumor suppressor by targeting angiotensin II type 1 receptor in pancreatic cancer.^19^ MiR-410 suppressed migration and invasion by targeting MDM2 in gastric cancer.^20^ These studies revealed that miR-410 was a tumor suppressor. Therefore, the functional roles of miR-410 in human cancers are cancer-type specific. Nevertheless, the functional importance of miR-410 and the molecular mechanisms in MM are still unclear.

In present study, we investigated the expression and biological role of miR-410 in MM progression. Our results showed that miR-410 was significantly upregulated in MM samples and cells for the first time. Its overexpression was associated with poor prognosis of MM patients. Gain- and loss-of-function experiment revealed that miR-410 promoted proliferation, cell cycle and apoptosis resistance of MM cells *in vitro* and *in vivo*. Notably, Krüppel-like factor 10 (KLF10) was identified as direct target of miR-410, resulting in activation of AKT signaling in cell proliferation and apoptosis. MiR-410 was inversely regulated by lncRNA OIP5-AS1. These results showed a novel role for miR-410 in predicting of prognosis and promoting tumor growth of human MM.

## Results

### miR-410 was significantly increased in MM samples and cells

**Figure 1:**
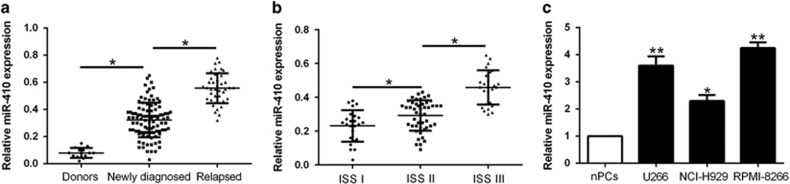
miR-410 is highly expressed in MM tissues and cell lines and correlates with the progression of MM. (**a**) Relative miR-410 expression levels in newly diagnosed, relapsed MM tissues and healthy donors were determined by qRT-PCR. (**b**) miR-410 level was compared between MM tissues of different ISS stage. (**c**) The expression of miR-410 in three MM cell lines was significantly increased compared to that in the nPCs cells. U6 snRNA was used as internal control. *P<0.05, **P<0.01

To investigate the potential role of miR-410 in MM, we first performed qRT-PCR to determine the miR-410 expression in 97 newly diagnosed MM samples and 14 healthy donors’ tissues. The data revealed that the mean level of miR-410 in MM tissues was significantly higher than that in normal plasma cells (*P*<0.01, Figure 1a). Moreover, we also observed that relapsed patients have higher miR-410 compared newly diagnosed patients (*P*<0.01, Figure 1a). Consistently, the expression of miR-410 in MM patients was also associated with ISS stage. The data revealed that the levels of miR-410 were increased at advanced stages (*P*<0.05, Figure 1b). Furthermore, we assessed miR-410 expression in MM cell lines and normal bone marrow-derived plasma cells (nPCs). All MM cell lines (RPMI-8266, U266 and NCI-H929) exhibited high expression as compared to nPCs (*P*<0.05, Figure 1c). These results indicated that miR-410 may be involved in the development of MM.

### Increased miR-410 is associated with poor survival in newly diagnosed MM patients

**Figure 2:**
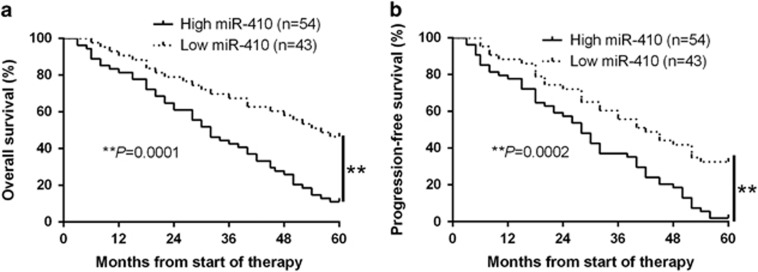
The prognostic value of miR-410 for MM patients assessed by Kaplan-Meier analysis. MM patients with high expression of miR-410 had worse (**a**) overall survival (OS) and (**b**) progression-free survival (PFS). **P<0.01

Since we have confirmed the correlation between high miR-410 expression and MM progression, we next explored the potential roles of miR-410 in MM patients’ clinical outcome. We determined 0.33 (mean level of miR-410) as a cutoff value for the expression level of miR-410. The expression of miR-410 was considered as either low or high. Kaplan-Meier survival curves suggest that patients with high miR-410 expression had remarkable shorter overall survival (OS, *P*=0.0001, Figure 2a) and progression-free survival (PFS, *P*=0.0002, Figure 2b) in newly diagnosed MM patients. These data suggest that miR-410 could be identified as a potential biomarker for the prognosis outcome of MM patients.

### miR-410 promotes cell proliferation, cell cycle progression and inhibits apoptosis in MM cells *in vitro* and *in vivo*

**Figure 3:**
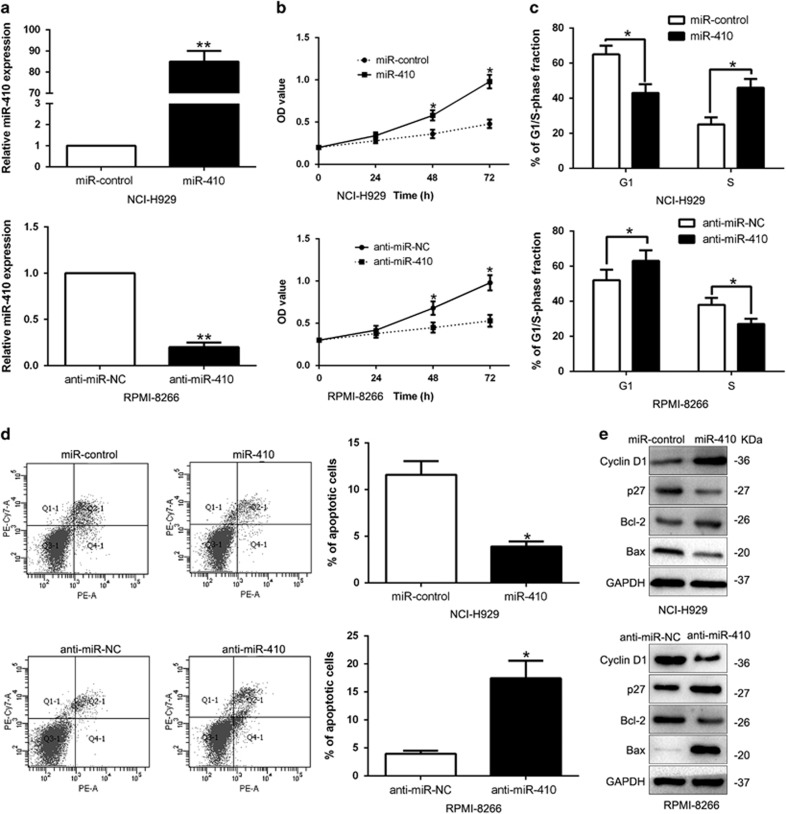
miR-410 promoted MM cell proliferation, cell cycle progression and inhibited cell apoptosis. (**a**) NCI-H929 cells and RPMI-8266 cells stably tranducedwith corresponding miRNA vectors were subjected to qRT-PCR for miR-410. Overexpression of miR-410 promoted cell proliferation (**b**), cell cycle progression (**c**) and inhibited apoptosis (**d**) in NCI-H929 cells, while down-regulation of miR-410 inhibited cell proliferation (**b**), cell cycle progression (**c**) and promoted apoptosis (**d**) in RPMI-8266 cells. (**e**) Western blot analysis of cycle regulator Cyclin D1 and p27, apoptosis-related protein Bcl2/Bax expression in the presence and absence of miR-410. *n*=6 independent experiments. *P<0.05, **P<0.01

To investigate the biological significance of miR-410 in MM, we stably overexpressing miR-410 in NCI-H929 cells by lentivirus system and stably knockdown miR-410 in RPMI-8266 cells which contained different endogenous miR-410 levels. As measured by qRT-PCR, we confirmed that miR-410 was effectively upregulated in NCI-H929 or downregulated in RPMI-8266 cells (*P*<0.01, Figure 3a). First, we assessed cell growth by CCK8 assays and found that miR-410 overexpression increased cell proliferation (*P*<0.05, Figure 3b). Next, as determined by flow cytometric analysis, the ectopic-expression of miR-410 promoted cell cycle transition from G1 to S phase (*P*<0.05, Figure 3c) and apoptosis resistance (*P*<0.05, Figure 3d). Furthermore, western blot confirmed that upregulated miR-410 markedly increased cycle-related protein, Cyclin D1 and apoptosis inhibition protein Bcl-2, while inhibited cycle inhibitor p27 and pro-apoptosis protein Bax (*P*<0.05, Figure 3e,Supplementary Figure 1). By contrast, miR-410 knockdown led to proliferation inhibition, G1 arrest and apoptosis promotion in RPMI-8266 cells (*P*<0.05, Figures 3b–e). These data demonstrated that miR-410 regulates the proliferation, cell cycle progression and apoptosis of MM cells in *vitro*.

**Figure 4:**
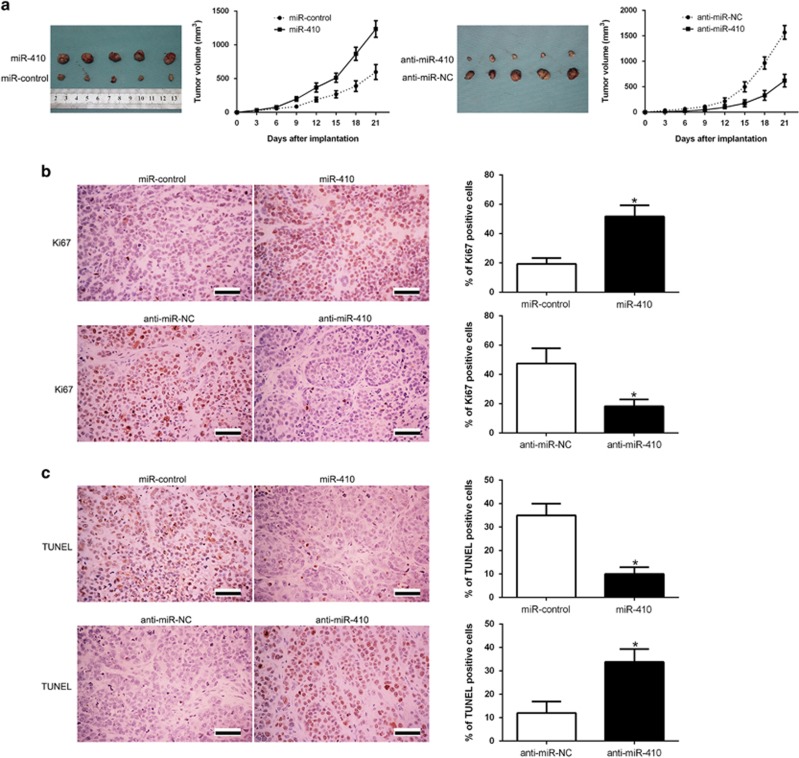
miR-410 promotes tumor growth and inhibits apoptosis *in vivo*. (**a**) Representative pictures of MM xenografts from both NCI-H929-miR-410 (left panel) and RPMI-8266-anti-miR-410 cells (right panel) (*n*=5). Tumor growth curve revealed that miR-410 overexpression significantly promoted, while miR-410 knockdown inhibited tumor growth *in vivo*. Tumor nodules were subjected to immunohistochemical staining for Ki-67 (**b**) and TUNEL (**c**) assays and quantitative analysis. Representative immunostaining and TUNEL assays revealed that miR-410 overexpression significantly increased the number of Ki-67 positive cells and inhibited the number of apoptotic cells. However, the percentage of Ki-67 positive cells in tumors arising from the miR-410 knockdown group was significantly lower and the percentage of apoptotic cells was significantly higher than that in the negative control (NC) group. *P<0.05

To further confirm the *in vitro* functional significance on MM cells, we establish the subcutaneous tumor model and the tumor growth curves revealed that miR-410 overexpression significantly promoted the tumor growth, while miR-410 knockdown retarded the tumor growth of MM cells in mice (*P*<0.05, Figure 4a). Next, we performed immunohistochemistry for Ki67 and TUNEL assays in the xenografted tissues. As expected, miR-410 overexpression increased the number of cells staining positive for Ki67 and reduced the number of apoptotic cells for TUNEL positive (*P*<0.05, Figures 4b and c). However, miR-410 knockdown led to a significant reduction in the number of proliferation cells staining for Ki67 and increased the number of apoptotic cells for TUNEL (*P*<0.05, Figures 4b and c). Taken together, these results demonstrated that miR-410 promoted tumor progression of MM *in vitro* and *in vivo*.

### KLF10 was a direct target of miR-410 in MM cells

**Figure 5:**
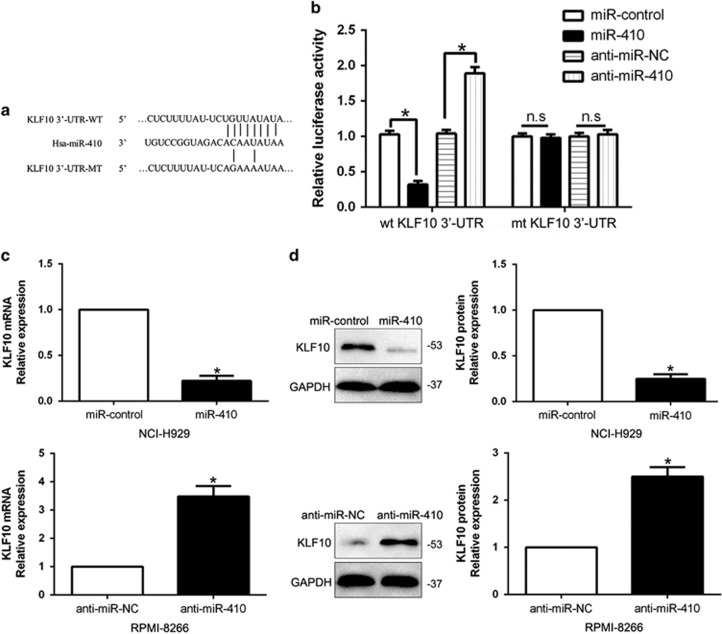
KLF10 was identified as a direct target of miR-410 in MM. (**a**) miR-410 and its putative binding sequence in the 3′-UTR of KLF10. The mutant binding site was generated in the complementary site for the seed region of miR-410. (**b**) miR-410 significantly suppressed the luciferase activity that carried wild-type (WT) but not mutant (MUT) 3′-UTR of KLF10. (**c**) qRT-PCR analysis of KLF10 mRNA expression in NCI-H929 cells with miR-410 or miR-control vector transfection and RPMI-8266 cells with anti-miR-410 or anti-miR-NC vector transfection. (**d**) Overexpression of miR-410 reduced the expression of KLF10 protein in NCI-H929 cells and knockdown of miR-410 increased the level of KLF10 protein in RPMI-8266 cells. *n*=six repeats with similar results, *P<0.05, **P<0.01

To explore the mechanism of miR-410 in MM progression, we used algorithm TargetScan to search candidate target of miR-410 and found that the 3′-UTR of KLF10 matched the 'seed sequence' of miR-410 (Figure 5a). To verify whether KLF10 was a direct target of miR-410 in MM cells, we carried out a luciferase reporter assay to confirm that miR-410 could bind to the 3′-UTR of KLF10. Reporter assays revealed that the increased miR-410 obviously inhibited the luciferase activity of wild-type (wt) KLF10 3′-UTR while had no influence on that of mutant (mt) KLF10 3′-UTR (*P*<0.05, Figure 5b). In contrary, the miR-410 reduction increased the luciferase activity of wt KLF10 3′-UTR (*P*<0.05, Figure 5b) but did not affect the luciferase activity of mt KLF10 3′-UTR constructs. Furthermore, miR-410 overexpression markedly suppressed the mRNA and protein expression of KLF10 in NCI-H929 cells (*P*<0.05, respectively, Figures 5c and d). By contrast, the expression of KLF10 mRNA and protein were significantly increased by the inhibition of miR-410 in RPMI-8266 cells (*P*<0.05, respectively, Figures 5c and d).

### miR-410 expression was inversely correlated with KLF10 in MM tissues

**Figure 6:**
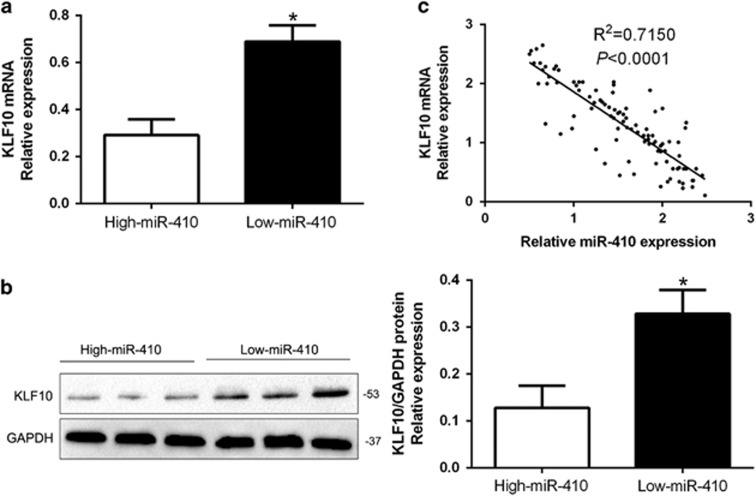
An inverse correlation between miR-410 and KLF10 expression is observed in MM. (**a**) The expression of KLF10 mRNA in miR-410 high-expressing tumors was significantly lower than that in miR-410 low-expressing tumors. (**b**) The expression of KLF10 protein in miR-410 high-expressing tumors was significantly lower than that in miR-410 low-expressing tumors. (**c**) A significant inverse correlation between the mRNA levels of KLF10 and miR-410 was observed in MM tissues. *P<0.05

To further investigate the relationship between miR-410 and KLF10 *in vivo*, we examined the mRNA and protein expression in diverse miR-410 expression groups. Results showed that KLF10 mRNA and protein levels were significantly lower in high miR-410 group than that in low miR-410 group in MM tissues (*P*<0.05, Figures 6a and b). In addition, we demonstrated that the mRNA level of KLF10 in the MM tissues was inversely correlated with miR-410 expression (*R*^2^=0.7150, *P*<0.0001, Figure 6c). In conclusion, these data suggest that KLF10 was a direct downstream target of miR-410 in MM.

### Alterations of KLF10 expression partially rescued the miR-410-induced biological effects on MM cells

**Figure 7:**
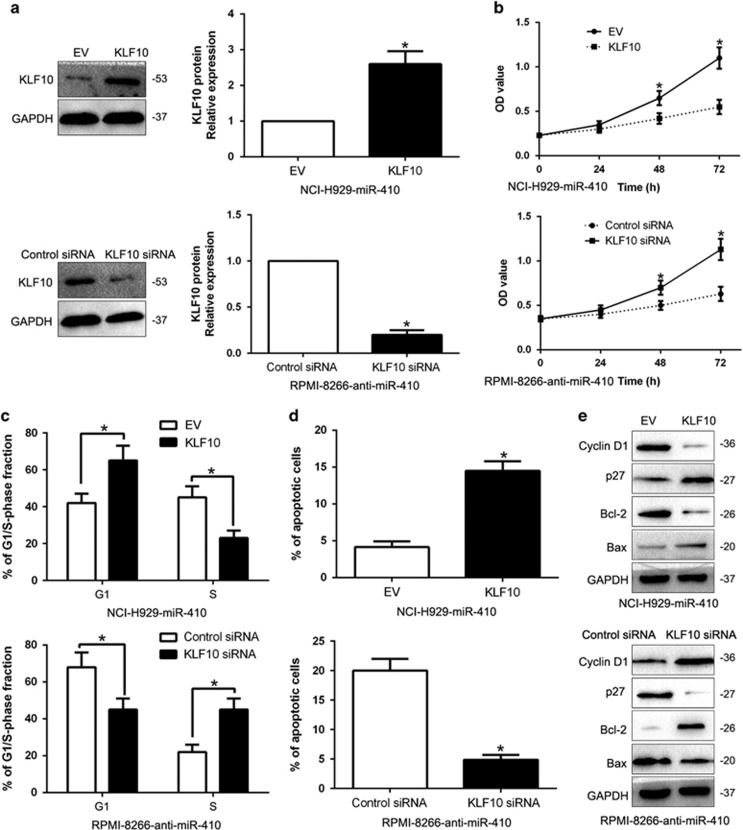
Alterations of KLF10 partially abolish miR-410-mediated MM cell proliferation, cell cycle progression and apoptosis. (**a**) miR-410-overexpressing NCI-H929 that were transfected with EV or KLF10 expression plasmid and miR-410-suppressive RPMI-8266 cells that were transfected with control siRNA or KLF10 siRNA were subjected to western blot analysis for KLF10. *n*=three repeats with similar results. The correlations between miR-410 effects and KLF10 knockdown or overexpression are shown in the (**b**) cell proliferation, (**c**) cell cycle progression and (**d**) apoptosis. KLF10 knockdown abrogated the effects of miR-410 knockdown on RPMI-8266 cells. KLF10 overexpression induced effects that were opposite to those stimulated by miR-410. (**e**) KLF10 restoration abrogated the effects of miR-410 on cell cycle and apoptosis-related regulators. *n*=3 independent experiments. *P<0.05, **P<0.01

To further confirm that KLF10 exerted its biological function as the target of miR-410, we restored KLF10 expression by overexpression construct plasmid in miR-410-overexpressing NCI-H929 cells (*P*<0.05, Figure 7a). Our data showed that KLF10 restoration partially abrogated the effect of miR-410, resulting in significant decrease of proliferation and promoted G1 phase arrest and apoptosis in miR-410-overexpressing NCI-H929 cells (*P*<0.05, Figures 7b–e). Similarly, silencing of KLF10 by a specific RNA interference in miR-410-suppressive RPMI-8266 cells partially abolished the effect of anit-miR-410 on cell proliferation, cell cycle progression and apoptosis (*P*<0.05, Figures 7a–e). These results demonstrate that KLF10 is a downstream mediator of miR-410 in MM.

### PTEN/AKT signaling plays substantial role in miR-410-mediated KLF10-induced biological function on MM cells

**Figure 8:**
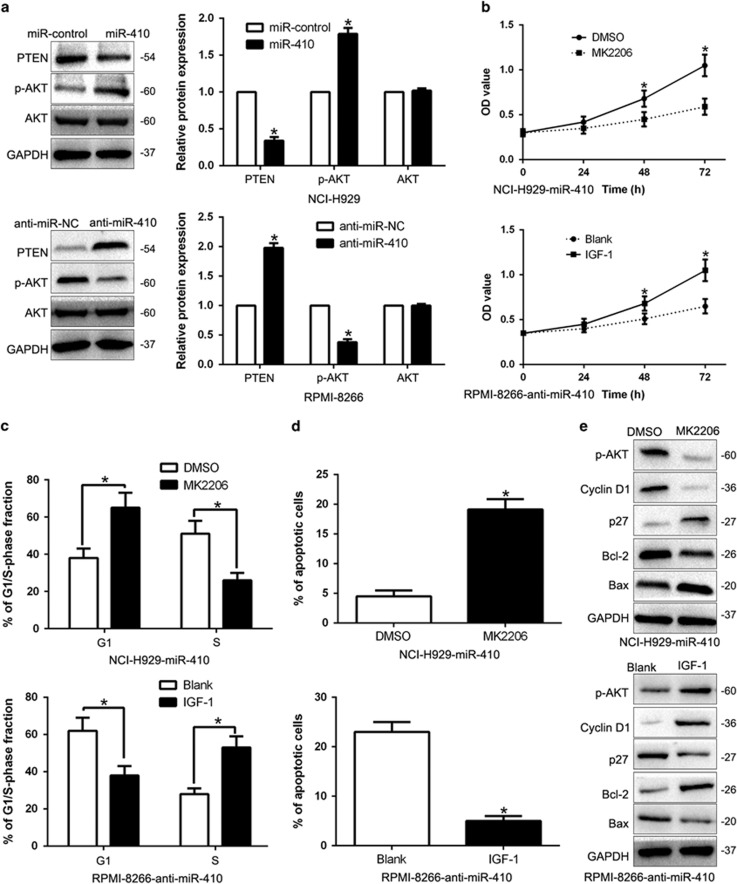
PTEN/AKT signaling is essential for the biological function of miR-410 in MM. (**a**) Western blotting analysis of PTEN, p-AKT (Ser473) and total AKT protein levels in NCI-H929-miR-410 or RPMI-8266-anti-miR-410 cells. Quantification of cell proliferation (**b**), cell cycle progression (**c**) and apoptosis (**d**) of RPMI-8266-anti-miR-410 treated with 100 ng/ml IGF-1 for 24 h or NCI-H929 cells stably expressing miR-410 treated with 1 *μ*M MK2206 for 24 h. (**e**) Western blot analysis of indicated proteins in RPMI-8266 cells stably expressing anti-miR-410 treated for 24 h with 100 ng/ml IGF-1or NCI-H929 cells stably expressing miR-410 treated with 1 *μ*M MK2206 for 24 h. *P<0.05

To explore the underlying mechanisms responsible for the pro-proliferation and anti-apoptosis effects in MM, we determined the PTEN/AKT signaling by western blot. As shown in Figure 8a, overexpression of miR-410 significantly increased, while miR-410 knockdown decreased the PTEN/AKT phosphorylation in MM cells (*P*<0.05, Figure 8a). but the total AKT protein had no change (*P*<0.05, Figure 8a,Supplementary Figure 2). Moreover, the downstream effectors of PTEN/AKT, Cyclin D1, p27, Bcl-2 and Bax were also significantly changed by the miR-410 expression (*P*<0.05, Figure 3e). These results revealed that miR-410 promoted the PTEN/AKT pathway in MM cells. To investigate whether AKT phosphorylation mediated miR-410-induced promotion of cell proliferation, cell cycle progression and apoptosis inhibition in MM cells, we treated miR-410-overexpressing NCI-H929 cells with the inhibitor of AKT phosphorylation MK2206. We found that MK2206 at least partially inhibited the miR-410-induced promotion of cell proliferation, cell cycle progression and apoptosis inhibition in MM cells (*P*<0.05, Figures 8b–e). Conversely, the insulin-like growth factor 1 (IGF-1), which is an activator of PTEN/AKT pathway, rescued the effects of miR-410 knockdown on cell proliferation, cell cycle progression and apoptosis inhibition (*P*<0.05, Figures 8b–e) in miR-410-suppressive RPMI-8266 cells. In conclusion, our results indicate that PTEN/AKT signaling plays an essential role in miR-410-mediated MM cell proliferation, cell cycle progression and apoptosis inhibition.

### miR-410 is negatively regulated by lncRNA OIP5-AS1 in MM cells

**Figure 9:**
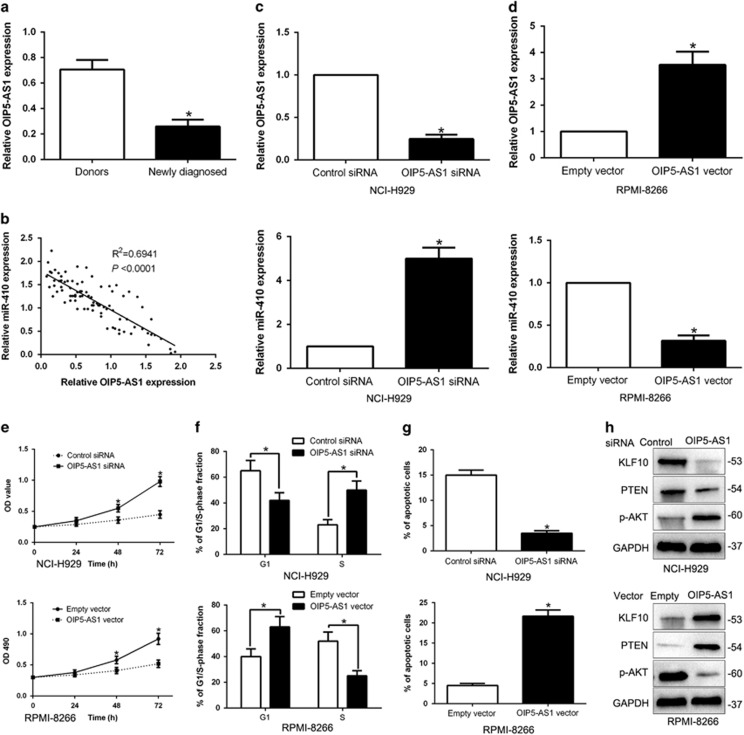
miR-410 is negatively regulated by lncRNA OIP5-AS1 in MM cells. (**a**) LncRNA OIP5-AS1 expression differences between MM tissues and healthy donors. *P<0.05. (**b**) An inverse correlation between the levels of miR-410 and OIP5-AS1 expression was observed in MM tissues. (**c**) NCI-H929 that were transfected with OPI5-AS1 siRNA or control siRNA were detected by qRT-PCR. OIP5-AS1 knockdown significantly increased the expression of miR-410 in NCI-H929 cells. *P<0.05. (**d**) RPMI-8266 cells that were transfected with OIP5-AS1 vector or empty vector were confirmed by qRT-PCR. OIP5-AS1 overexpression notably reduced the expression of miR-410 in RPMI-8266 cells. Overexpression of OIP5-AS1 inhibited cell proliferation (**e**), cell cycle progression (**f**) and promoted apoptosis (**g**) in RPMI-8266 cells, while down-regulation of OIP5-AS1 promoted cell proliferation (**e**), cell cycle progression (**f**) and inhibited apoptosis (**g**) in NCI-H929 cells. (**h**) Western blot analysis of downstream KLF10/PTEN/AKT protein in the presence and absence of OIP5-AS1. *n*=6 independent experiments. *P<0.05

To investigate the reason for miR-410 was increased in MM, we predicted a target by Starbase 2.0 and found lncRNA OIP5-AS1 is a molecular sponge that modulates miR-410. Next, our data revealed the levels of OIP5-AS1 in MM tissues were notably lower than those in healthy donors (*P*<0.05, Figure 9a). Spearman correlation analysis revealed that the levels of OIP5-AS1 were inversely correlated with miR-410 expression in MM tissues (R^2^=0.6941, *P*<0.05, Figure 9b). Furthermore, OIP5-AS1 expression was knocked down by a specific siRNA in NCI-H929 cells and OIP5-AS1 knockdown increased the expression of miR-410 in NCI-H929 cells (*P*<0.05, Figure 9c). Nevertheless, OIP5-AS1 overexpression led a reduction of miR-410 in RPMI-8266 cells (*P*<0.05, Figure 9d). Then, we investigated whether OIP5-AS1 regulated biological function and KLF10 expression and PTEN/AKT signaling in MM cells. As expected, OIP5-AS1 knockdown promotes cell proliferation, cell cycle progression and inhibits apoptosis (*P*<0.05, Figures 9e–g). Interestingly, OIP5-AS1 knockdown inhibited KLF10 expression and PTEN/AKT progression in NCI-H929 cells (Figure 9h). On the contrary, OIP5-AS1 overexpression inhibited cell proliferation, cell cycle progression and induced apoptosis, and promoted KLF10 expression and PTEN/AKT process in RPMI-8266 cells (Figures 9e–h). Thus, lncRNA OIP5-AS1 contributes to KLF10/PTEN/AKT signaling pathway in MM cells possibly by negatively regulating miR-410.

## Discussion

Increasing evidence has demonstrated that abnormal miRNAs play critical role in the cancer initiation, development and progression of MM, which has been emphasized as valuable diagnostic and prognostic biomarker and attractive therapeutic targets of MM. However, the clinical significance and molecular mechanisms of specific miRNA are still needed to be investigated. Here, we demonstrated for the first time that the expression of miR-410 was significantly upregulated in newly diagnosed and relapsed patients than healthy donors. Moreover, our data suggest that increased miR-410 expression was positively associated with advanced ISS stage. In addition, we provided the first evidence that overexpression of miR-410 conferred an obvious poor prognosis of MM patients. Therefore, these data confirmed that miR-410 potentially functions as a prognostic marker in MM.

Previous studies reported that miR-410 is overexpressed in liver and colorectal tumors and enhances tumor cell growth by silencing FHL1 via a methylation-related direct/indirect mechanism.^21^ MiR-410 induces stemness by inhibiting Gsk3*β* but upregulating *β*-catenin in non-small cells lung cancer.^22^ However, miR-410 regulates MET to influence the proliferation and invasion of glioma.^23^ MiR-410 suppresses cell proliferation and invasion of osteosarcoma by targeting VEGF.^24^ Therefore, the expression status and role of miR-410 in human cancers is a controversial topic. In the present study, we disclosed the biological function of miR-410 in MM. We demonstrated that miR-410 overexpression promoted cell proliferation, cell cycle progression and apoptosis inhibition of NCI-H929 cells, while miR-410 knockdown facilitated these cellular behaviors of RPMI-8266 cells *in vitro*. In addition, miR-410 overexpression promoted, while miR-410 knockdown inhibited tumor growth in subcutaneous tumor model. These data revealed that miR-410 functions as an oncogene by regulating proliferation, cell cycle and apoptosis in MM cells.

KLF10, originally called TGF-*β* inducible gene 1 (TIEG1), has been found to be downregulated in human cancers and inhibits growth, radio-sensitivity and metastasis of cancer cells.^25, 26, 27, 28^ Here, we confirmed that KLF10 was a direct downstream target of miR-410 based on the following reasons: firstly, luciferase activity assays indicated that miR-410 could bind with the 3′-UTR of KLF10. Next, we found miR-410 inversely regulated KLF10 abundance in MM cells and an inverse correlation between miR-410 and KLF10 expression was observed in MM tissues. Moreover, KLF10 restoration abrogated the effects of miR-410 on the proliferation, cell cycle and apoptosis of MM cells. Previous studies confirmed that KLF10 could regulate the PTEN/AKT signaling pathway.^29, 30^ Here, we disclosed that miR-410 promoted cellular behaviors through PTEN/AKT pathway-mediated cell cycle regulator Cyclin D1 and p27, apoptosis-related Bcl-2/Bax expression. The AKT activation could influence the effects of miR-410 on MM cells. These results suggest the exact role of miR-410 in MM. Furthermore, we explored the reason for miR-410 overexpression in MM. Previous studies reported that miR-410 is regulated by lncRNA expression.^31^ Here, we identified lncRNA OIP5-AS1 was downregulated in MM tissues compare to healthy donors, and was inversely correlated miR-410 expression in MM tissues. Next, we demonstrated that OIP5-AS1 inversely regulated miR-410 expression and promotes KLF10-mediated PTEN/AKT signaling in MM cells. Taken together, the OIP5-AS1-miR-410-KLF10/PTEN/AKT signaling axis probably exerts key functions in the cell proliferation, cell cycle progression and apoptosis inhibition of MM and may represent a therapeutic target for MM patients.

In conclusion, we show that miR-410 acts as an oncogene in MM. Firstly, our results demonstrate that miR-410 expression was upregulated in MM tissues and cell lines. Then, our clinical data suggest that miR-410 may be used as a novel prognostic marker for MM patients. Moreover, loss of lncRNA OIP5-AS1 induced miR-410 accumulation facilitates cell proliferation, cell cycle progression and apoptosis inhibition via targeting KLF10/PTEN/AKT signaling in MM cells. Taken together, our results verify that miR-410 may be served as a potential target for cancer therapeutics in MM.

## Materials and methods

### Clinical specimens

97 MM tissues and 14 healthy donors’ samples were collected from Department of Hematology, the Second Affiliated Hospital of Xi’an Jiaotong University during January 2004 to December 2011. The monoclonal component was IgG in 35 cases, IgA in 26 cases, IgD in 2 cases, IgM in 2 cases, light chain 30 cases and no secretion in 2 cases. All patients were diagnosed based on World Health Organization diagnostic criteria of multiple myeloma. The normal bone marrows from healthy donors were collected as controls. Mononuclear cells (MNCs) were isolated from BM aspirates of MM patients by Ficoll-Hipaque (Pharmacia, Piscataway, NJ, USA) density sedimentation. CD138+ cells were selected from MNCs using EasyStep CD138+ magnetic nanoparticles, as described in the instructions from the manufacturer’s protocol (Stem Cell Technologies, Vancouver, BC, Canada). The percentage of CD138+ cells isolated from bone marrow of normal donors was 0.5-2% in mononuclear cells. The purity of the cell preparation was verified to be 95% by fluorescence-activated cell sorting (FACS) analysis and light microscopy. All patients had written informed consent and this research was approved by the Ethical Committee of Xi'an Jiaotong University.

The human MM cell lines NCI-H929, U266 and RPMI-8266 and the normal plasma cells (nPC) were cultured in RPMI-1640 (Gibco, Carlsbad, CA, USA) containing 10% FBS (Invitrogen, Carlsbad, CA, USA), 1% penicillin-streptomycin (Sigma, St. Louis, MO, USA) in a humidified atmosphere at 37 °C with 5% CO2.

### Quantitative reverse transcriptase PCR

Total RNA from MM tissues and cells was isolated using TRIzol reagent (Invitrogen, Carlsbad, CA) according to the manufacturer’s protocol. cDNA was reverse-transcribed from 2 *μ*g total RNA using a Reverse Transcription Kit (Takara, Biochemical, Tokyo, Japan). cDNA was then amplified with a SYBR Premix Ex Taq II (Perfect Real-Time) kit (Takara). The gene expression levels were calculated using the delta-delta Ct method with U6 or GAPDH as an internal control. Hsa-miR-410 primer (HmiRQP0485), snRNA U6 qPCR Primer (HmiRQP9001), KLF10 (HQP018084) and GAPDH (HQP006940) were purchased from Genecopoeia (Guangzhou, China).

### Lentivirus transduction and oligonucleotide transfection

The lentiviral particles for miR-410 overexpression and inhibition constructs were packaged and purchased from GeneChem (Shanghai, China). NCI-H929 and RPMI-8226 were infected with recombinant lentivirus transducing units plus 5 *μ*g /ml Polybrene (Sigma, Natick, MA, USA). Cells were collected 48 h after transduction.

LncRNA OIP5-AS1 vectors, including OIP5-AS1 expression vector, the empty vector, OIP5-AS1 siRNA and control were synthesized and purchased from by Shanghai Genepharm Co. Ltd (Shanghai, China). The KLF10 overexpression plasmid and specific siRNA against KLF10 and a scramble siRNA were synthesized by Sangon Biotech Co., Ltd. (Shanghai, China). Cells were seeded at a concentration of 2 × 10^6^ per well in six-well plates and transfected with 100 nm above vectors using Lipofectamine 2000 Reagent (Invitrogen Life Technologies) and combination with electroporation system in accordance with the manufacturer's protocol.

### Western blot analysis

The whole proteins were lysed in RIPA buffer supplemented with protease and phosphatase inhibitors (Roche) and the concentrations were quantified with BCA Protein Assay Kit (Tiangen, Beijing, China), and an equal amount of 40 *μ*g protein was separated by 10% SDS-PAGE gel and then transferred onto PVDF membranes (Millipore, Billerica, MA, USA). The membranes were blocked with 5% nonfat milk in TBST for 2 h at room temperature and incubated overnight with KLF10 antibodies (1:1000, Abcam, Cambridge, MA, USA) at 4 °C. Then the membranes were washed three times by TBST and incubated with HRP-conjugated secondary antibody for 2 h at room temperature (ZSGB-BIO, China). Detection was performed by enhanced chemiluminescence kit (Amersham, Little Chalfont, UK). GAPDH was used as protein loading control. The antibodies against PTEN, Cyclin D1, p27, AKT, p-AKT, Bcl-2 and Bax were purchased from Cell Signaling Technology (Danvers, MA, USA). The intensity of protein bands was quantified using Quantity One software 4.5.0 basic (Bio-Rad, Hercules, CA, USA).

### Cell proliferation, cell cycle and apoptosis detection

For the proliferation assay, cells were seeded in 96-well plate at the density of 5 × 10^3^ cells/well in 90 *μ*l volume and Cell Counting Kit-8 (CCK8) reagents (Dojindo, Kumamoto, Japan) were used according to the manufacturer’s instruction. Flow cytometry was performed using the fluorescence-activated cell sorting (FACS) Calibur and Cell Quest software (both from Becton-Dickinson, San Jose, CA, USA). For cell cycle assay, the cells were seeded in 6-well plates at 2 × 10^5^/well. 48 h after transfection, the cells were fixed in 70% ethanol at 4 °C for 24 h and stained with 50 *μ*g/ml propidium iodide (Keygen, Nanjing, China). An Annexin-V-Fluos Staining kit (Roche, Basel, Switzerland) was used to analyze apoptosis levels.

### Luciferase reporter assay

The 3′-UTR sequence of KLF10 predicted to interact with miR-410, together with a corresponding mutated sequence within the predicted target sites, were synthesized and inserted into the pmiR-GLO dual-luciferase miRNA target expression vector (Promega, Madison, WI, USA) called wt KLF10 3′-UTR and mt-KLF10 3′-UTR. Subsequently, HEK293 cells that were plated into 24-well plate and were transfected with miR-410 inhibitor or negative control. Cells were co-transfected with the wild-type or mutant 3′-UTR of KLF10 vector using the Lipofectamine 2000 reagent (Invitrogen). After 48 h, cells were harvested and measured according to the manufacturer’s instructions (Dual-Luciferase Assay System; Promega). pRL-TK expressing Renilla luciferase was cotransfected as an internal control to correct the differences in both transfection and harvest efficiencies.

### *In vivo* experiments

Four-to-six-week-old female BALB/c nude mice (Centre of Laboratory Animals, The Medical College of Xi'an Jiaotong University, Xi'an, China) were used to establish the nude mouse xenograft model. NCI-H929 (5 × 10^6^) cells that were transduced with miR-410 or miR-control vectors or RPMI-8266 cells with anti-miR-410 were mixed in 150 *μ*l of Matrigel and were inoculated subcutaneously into the flank of nude mice. The tumor volume for each mouse was determined by measuring two of its dimensions and then calculated as tumor volume=length × width × width/2. After 3 weeks, the mice were sacrificed by cervical dislocation under anesthesia with ether and the xenograft tumor tissue was explanted for examination. Animal protocols were approved by the Institutional Animal Care and Use Committee of Xi'an Jiaotong University.

### Statistical analysis

Data are presented as the mean±SD and performed at least three independent replicates. SPSS software, 16.0 (SPSS, Inc., Chicago, IL, USA) and Graphpad Prism 6.0 (San Diago, CA, USA) were used for a two-tailed Student's *t*-test, Pearson's correlation analysis, Kaplan–Meier method and the log-rank test to evaluate the statistical significance. Differences were defined as *P*<0.05.
