# Supplementary figures and images for: LncRNA OIP5-AS1 loss-induced microRNA-410 accumulation regulates cell proliferation and apoptosis by targeting KLF10 via activating PTEN/PI3K/AKT pathway in multiple myeloma
Source: Cell Death Dis. 2017 Aug 10;8(8):e2975–. doi: 10.1038/cddis.2017.358 (PMC5596549; doi:10.1038/cddis.2017.358)

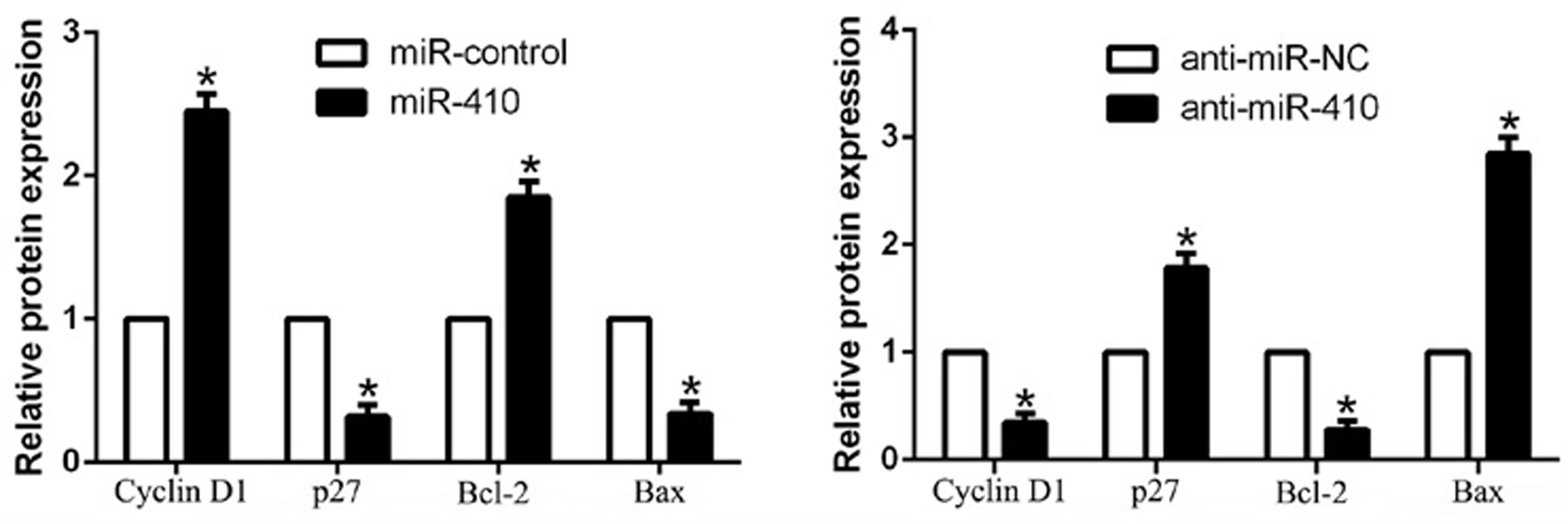

Supplement: Supplementary Figure 1 [file cddis2017358x1.tif]

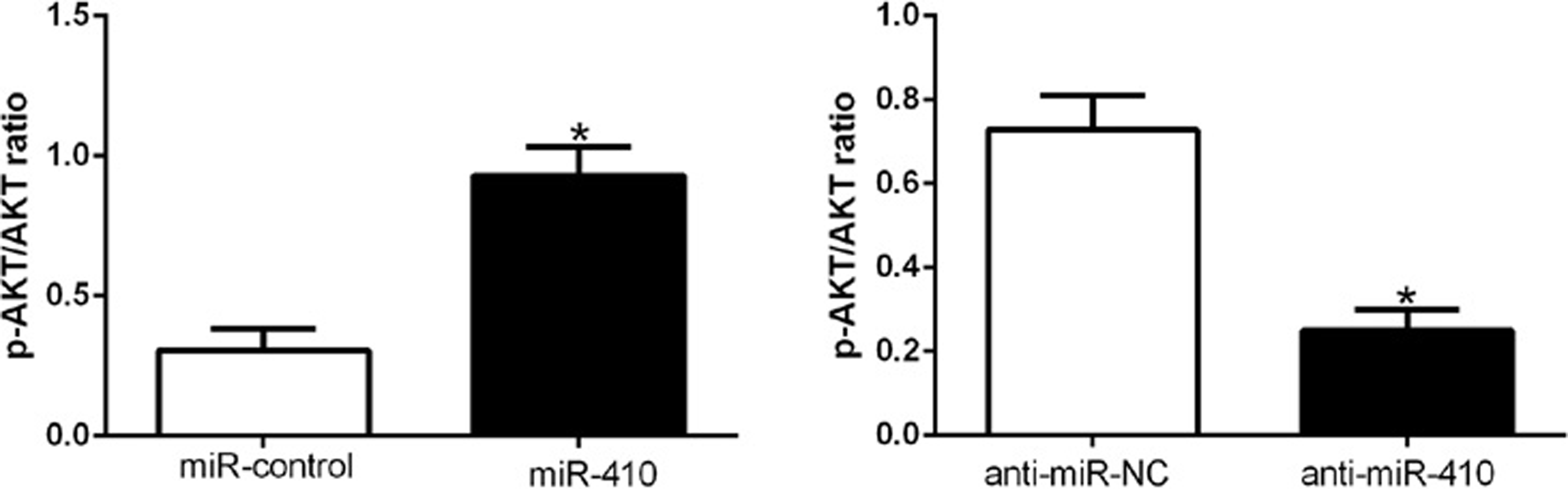

Supplement: Supplementary Figure 2 [file cddis2017358x2.tif]
