# Supplementary material for: LncRNA OIP5-AS1 loss-induced microRNA-410 accumulation regulates cell proliferation and apoptosis by targeting KLF10 via activating PTEN/PI3K/AKT pathway in multiple myeloma
Source: Cell Death Dis. 2017 Aug 10;8(8):e2975–. doi: 10.1038/cddis.2017.358 (PMC5596549; doi:10.1038/cddis.2017.358)
Supplement: Supplementary Figure Legends [file cddis2017358x3.docx]

**LncRNA OIP5-AS1 loss-induced microRNA-410 accumulation regulates cell proliferation and apoptosis by targeting KLF10 via activating PTEN/PI3K/AKT pathway in multiple myeloma**

**Supplementary Figure Legends**

**Supplementary Figure 1:** Relative densitometric analysis of cycle-related protein, Cyclin D1, p27 and apoptosis-inhibition protein Bcl-2, Bax after miR-410 overexpression or down-regulation. *P<0.05.

**Supplementary Figure 2:** Relative densitometric analysis of the ration of p-AKT/AKT expression after miR-410 overexpression or down-regulation. *P<0.05.
